# Anticholinergic burden in middle and older age is associated with reduced cognitive function, but not with brain volume.

**DOI:** 10.23889/ijpds.v7i3.1821

**Published:** 2022-08-25

**Authors:** Jure Mur, Riccardo Marioni, Tom Russ, Graciela Muniz-Terrera, Simon Cox

**Affiliations:** 1 University of Edinburgh

## Objectives

Anticholinergic drugs block muscarinic receptors in the body. They are commonly prescribed for a variety of indications and their use has previously been associated with dementia and cognitive decline

## Approach

In UK Biobank participants with linked health-care records (n=171,266, aged 40-71 at baseline), we calculated total anticholinergic drug burden according to 15 different anticholinergic scales and due to different classes of drugs. We then used linear regression to explore the associations between anticholinergic burden and various measures of cognition and structural MRI, including general intelligence, 9 separate cognitive domains, total brain volume, volumes of 68 cortical and 16 subcortical areas, and fractional anisotropy and median diffusivity of 25 white-matter tracts.

## Results

Anticholinergic burden was modestly associated with poorer cognition across most anticholinergic scales and cognitive tests (6/9 FDR-adjusted significant associations, std. betas range: -0.033, -0.006). The association was mostly driven by antibiotics (std. beta=-0.029, 95% p<0.001) and drugs to treat disorders of the nervous system (std. beta=-0.017, p<0.001). Anticholinergic burden due to the pharmacological subclass of glucose-lowering drugs (beta=-0.038, p<0.001) and the anatomical class of respiratory drugs (beta=0.016, p=0.03) was associated with total brain volume. However, anticholinergic burden was not associated with any other measure of brain macro- or microstructure (p>0.07).

## Conclusion

Anticholinergic burden is mildly associated with poorer cognition, but there is little evidence for an effect for measures of brain structure. Future studies might focus more broadly on polypharmacy or more narrowly on distinct drug classes, instead of using purported anticholinergic action to study the effects of drugs on cognitive ability.

